# Application of Focus Variation Microscopy and Dissolution Imaging in Understanding the Behaviour of Hydrophilic Matrices

**DOI:** 10.3390/pharmaceutics12121162

**Published:** 2020-11-28

**Authors:** Adam Ward, Benedict Brown, Karl Walton, Peter Timmins, Barbara R. Conway, Kofi Asare-Addo

**Affiliations:** 1 Department of Pharmacy, University of Huddersfield, Huddersfield HD1 3DH, UK; Adam.Ward@hud.ac.uk (A.W.); Benedict.Brown@hud.ac.uk (B.B.); p.timmins@hud.ac.uk (P.T.); b.r.conway@hud.ac.uk (B.R.C.); 2 EPSRC Future Metrology Hub, University of Huddersfield, Huddersfield HD1 3DH, UK; K.Walton@hud.ac.uk

**Keywords:** hypromellose, xanthan gum, polyethylene oxide, surface dissolution imaging, focus variation, swelling

## Abstract

Hydrophilic matrix systems can be found in a wide range of extended release pharmaceutical formulations. The main principle of these systems is that upon contact with water, the hydrophilic component swells to form a hydrated gel layer which controls drug release. The following work demonstrates an explorative study into the use of dissolution imaging and focus variation microscopy with hydrophilic polymers. This study investigated the surface properties of xanthan gum (XG), polyethylene oxide (PEO), and hypromellose (hydroxypropyl methylcellulose, HPMC) compacts with each of these three hydrophilic polymers from one of each classification of natural, semi-synthetic, or synthetic polymer using a focus variation instrument. The auto correlation length (S*al*) showed all surface profiles from the compacts displayed a value below 0.1 mm, indicating that only high frequency components (i.e., roughness) were considered and that the analysis had been successful. The developed interfacial area ratio (S*dr*) displayed values below 5% in line with ISO guidelines for all the polymers studied with their texture aspect ratio values (S*tr*) > 0.5, indicating uniformity of the surfaces of the produced compacts. Of the various parameters studied, areal material ratio (S*mr*2) predicted XG to wet and hydrate quicker than PEO, with PEO also wetting and hydrating quicker than the HPMC. The dissolution imaging and initial swelling studies proved to concur with the findings from the areal material ratio (S*mr*2) parameter, suggesting porosity was not an indicator for the ease with which water ingress occurs. This study suggests the S*mr2* surface parameter to potentially predict wetting and initial hydration of hydrophilic polymers, however care should be taken as this study consists of a selected number of hydrophilic polymers.

## 1. Introduction

Hydrophilic matrix systems can be found in a wide range of extended release (ER) pharmaceutical formulations. There are many advantages to the use of hydrophilic matrix technology, such as an existing knowledge base, regulatory status, and low cost of polymers used and ease of manufacture [1]. They provide for a number of key therapeutic benefits, including reduction in dosing frequency, possibility of mitigation of side effects, and improved patient compliance. Potential limitations include drug release from the matrices being compromised by food and gastric transit and that dosing of larger matrix tablets may be difficult as dosage form cannot be crushed or chewed to facilitate administration.

The main release rate controlling principle of these systems is that upon contact with water, the hydrophilic component swells to form a dynamic hydrated gel layer around the dosage form. This gel layer evolves over time through the further ingress of fluid, the rate of further water penetration into the tablet core facilitating the control of diffusional release of drugs [2]. Erosion of the hydrated polymer under shear at the interface of the gel layer and the immersion medium also can contribute to drug release rate control. The dissolution rate of soluble drugs is principally controlled by diffusion out of the hydrated gel layer, whilst the dissolution rate of poorly soluble drugs is controlled by the rate of erosion of the hydrated gel layer. The rate of erosion depends on the type of polymer used within the formulation [3,4].

A wide range of hydrophilic polymers are available for use in ER formulations. These can be classified in a number of ways based on their structure, degradability, or chemistry. However, one of the most common ways of classifying the polymers is based on their source e.g., natural or synthetic. This work focuses on three polymers representing each classification. Xanthan gum (XG) is a natural high molecular weight anionic polysaccharide that is obtained through the fermentation of carbohydrates with *Xanthamonas campestris* [5]. XG has been widely used in oral and topical formulations as well as in the food and cosmetic industry as a stabilising or suspending agent. It has also been used as a hydrophilic matrix [6,7]. Polyethylene oxide (PEO) is a synthetic thermoplastic homopolymer synthesised by the heterogeneous catalytic polymerisation of the ethylene oxide monomer [8]. PEO is available in a wide range of molecular weights and due to the oxide present in the polymer chain, it is highly soluble in water. PEO has been utilised in a number of ER applications and been shown to control the release of both high and low solubility drugs from matrix formulations manufactured via direct compression [9,10,11]. PEO has also been used in other formulations and applications including buccal adhesives [12], hydrogels [13], ER capsules [14], and hot-melt extruded tablets [15]. Hydroxypropyl methylcellulose (hypromellose, HPMC) is a semi-synthetic hydrophilic polymer and is commonly used to control drug release. Amongst the wide range of hypromellose grades available, the high molecular weight hypromellose 2208, USP grade is the most widely used in ER matrix formulations [16]. There are several imaging techniques reported that have been used to aid the understanding of drug release from hydrophilic matrix tablets. Some of these imaging techniques include near infra-red (NIR), magnetic resonance imaging (MRI), nuclear magnetic resonance (NMR), terahertz, confocal laser scanning microscopy (CLSM), UV (vis) imaging and attenuated total reflection-Fourier transform infrared (ATR-FTIR) imaging [4,17,18,19,20,21,22,23,24,25,26,27,28,29,30,31,32].

UV imaging has been used to report several phenomena, including intrinsic dissolution rate events, permeation through synthetic membranes, films for buccal delivery, and swelling in one sided 3 mm hydrophilic compacts [33,34,35,36,37,38,39,40,41,42,43]. Ward et al. recently developed a material sparing methodology using dissolution imaging (surface dissolution imaging instrument (SDI2)) for determining the swelling of whole compacts using hypromellose as a model hydrophilic matrix former [3]. The developed methodology successfully allowed the initial swelling behaviour of hypromellose to be investigated by utilising a lower absorbance of 50 mAu with a wider measurement zone in determining the edge of the gel layer and growth in real-time. This current work aims to test the developed methodology on a natural polymer (XG) and synthetic polymer (PEO) and semi-synthetic polymer (HPMC controlled release grade (CR)) to determine if the reported parameters of using the lower absorbance of 50 mAu as well as wider measurement zone as reported in [3] does indeed work for synthetic and natural polymers.

**Figure 1:**
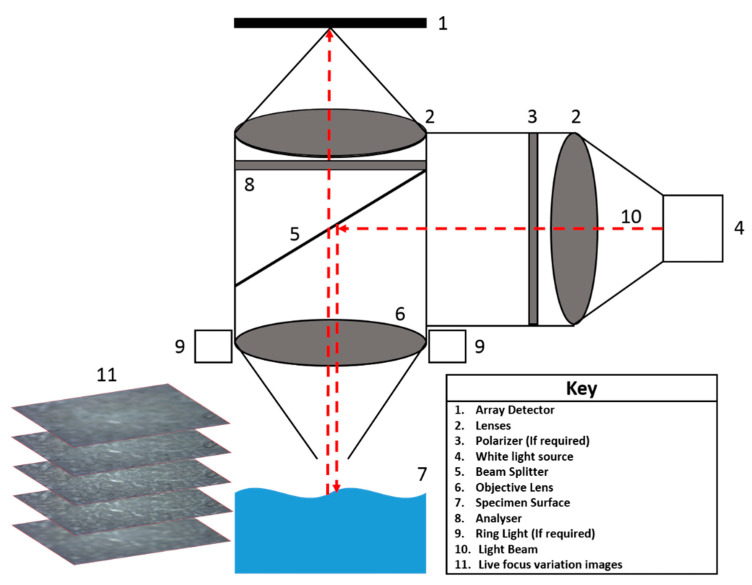
A schematic representation of a focus variation microscope and how this system varies focus to obtain topography information.

To characterise surfaces effectively, 3D measurements are necessary. This is because they provide a more detailed analysis of the surface textural properties, and therefore information on how the surfaces can impact an investigation [44,45]. A focus variation instrument (FVI) is proposed as the instrument (Alicona) used in characterising the surfaces of the compacts under investigation. Focus variation microscopy is an optical 3D micro coordinate measurement system for complete form measurement of cutting edges [46,47]. Form, contour, and roughness are measured in high resolution in areal (2.5D) and three-dimensional (Real3D) perspectives. Focus variation combines the small depth of focus of an optical system with vertical scanning (Figure 1). This allows for the provision of topographical and colour information from the variation in focus [48]. The FVI has many applications, especially in quality assurance in micro-precision in engineering in the aerospace sector, [49] but is beginning to be used in the field of pharmaceutics for understanding punch surfaces for tabletting as well as surfaces for compacts for intrinsic dissolution measurements [33,36,37,45].

To understand surface texture measurements, it is important to understand profile and areal surface measurement. Surface profile measurement is the measurement of a single line across the surface which can be represented mathematically as a height function with lateral displacement (z(x)). Areal surface texture measurement is described as the measurement of an area on the surface that can be defined mathematically as a height function with displacement across a plan (z(x,y)). Areal surface measurement offers many benefits over profile measurement, including a more realistic representation of the whole surface with greater statistical significance, a lower chance of significant surface features being missed, and provides a better visual record of the overall structure of the surface [46]. All surface texture measurements fall under the scope of the International Organisation for Standardisation (ISO) and as a result, a number of specification standards have been released governing both profile and areal measurement (Table 1) [50]. Four carefully selected key surface parameters are selected from the ISO which is of interest to this reported work and discussed in the Methodology section (Section 2.2.5). Another aim of this study was to use these parameters in understanding the surfaces produced after the compaction of the hydrophilic polymers and investigating possible links to the early wetting/gel layer formation associated to the dissolution process. To the best of our knowledge, there is no reported work combining the techniques of focus variation and dissolution imaging in understanding this phenomenon.

## 2. Materials and Methods

### 2.1. Materials

Xanthan gum (XG) (XANTURAL^®^ 75) was kindly provided by CP Kelco Ltd. (Leatherhead, UK) and had a molecular weight of approximately 2,000,000 Da [51,52]. Polyethylene oxide (PEO) MW 4,000,000 (POLYOX^™^) and hydroxypropyl methylcellulose (HPMC) 2208, 100,000 cps grade (Methocel K100M CR) were kind gifts from Colorcon, Dartford, UK. Deionised water was used as the dissolution media for all hydration studies.

### 2.2. Methods

#### 2.2.1. True Density

The true density measurements were performed on the three polymers of interest. Briefly, approximately 2 g of powder was added to a stainless steel sample cup. The sample cup was then placed in an Accupyc II gas pycnometer and sealed. Helium gas was used as the inlet gas. Each measurement took approximately 10 min and were conducted in triplicate.

#### 2.2.2. Compact Manufacture

10 mm flat faced compacts of approximately 312.5 ± 1 mg of each polymer under investigation was weighted out and compressed at 10 kN using a Testometric™ hydraulic press. The compacts were ejected from the die and stored in a glass vial at room temperature prior to use. Only pure polymer compacts were investigated in this study.

#### 2.2.3. Tablet Dimensions, Hardness and Porosity

Five compacts for each polymer (XG, PEO, and HPMC) were used for measuring tablet dimensions and hardness. The weight of each compact was measured prior to placement in a hardness tester (PharmaTest, Hainburg, Germany). The porosity was then determined as the difference between the apparent density (*ρ_app_*) of the compact and the true density of the powder (*ρ_true_*) (Equation (1)):

(1) $$Porosity=\left(\left(\frac{\rho_{app}}{\rho_{true}}\right)-1\right)\;\times 100.$$

#### 2.2.4. Scanning Electron Microscopy (SEM)

Prior to analysis on the SEM, each polymer sample was mounted on double-sided carbon adhesive tape on a metal stub and sputter-coated with a thin coating of gold/palladium (80:20) for 60 s using a Quorum SC7620 Sputter Coater under vacuum. A Jeol JSM-6060CV SEM operating at 20 kV was used to obtain the electron micrographs. Different magnifications were taken to aid the study of particle morphology.

#### 2.2.5. Surface Assessment of the Compact Using Focus Variation Microscopy

Prior to dissolution, imaging surface assessment of the manufactured compacts was conducted for all the three polymers using the focus variation instrument (Alicona). Four carefully selected parameters from the ISO guidance were used in the assessment of the surfaces produced post the compaction process reported in Section 2.2.2.

Developed Interfacial Area Ratio (S*dr*)

The true surface area of a textured sample compared to that of a uniform flat surface of equal size is known as the S*dr.* This was determined for each of the compacts from all three polymers (XG, PEO, and HPMC). The S*dr* can be expressed as a percentage or as a unit less positive number by which the true measured surface area exceeds that of the nominal uniform measurement area (Equation (2)). Typically, the parameter produces a value of between 0 and 10% for most surfaces, with 0% representing a completely smooth surface:

(2) $$Sdr=\;\frac{\left(Texture\;Surface\;Area\right)\;-\;\left(Cross\;Sectional\;Area\right)}{Cross\;Sectional\;Area}$$

Auto Correlation Length (S*al*)

S*al* is a measurement of the lateral scale of a surface and is defined as the horizontal distance of the autocorrelation function (ACF) (dx,dy) which has the fastest decay to a specified value s, where s is between 0 and 1 (Equation (3)). In this work, a value of 0.2 was set in line with guidance described in ISO 25178 [50]. S*al* in this work was, however, used as a confirmatory tool to ensure that the surface analysis had been conducted correctly and only surface roughness was considered in all surface calculations. A large value for S*al* indicates that a surface is dominated by low frequency components i.e., waviness, whereas a low value for S*al* denotes the opposite i.e., roughness:

(3) $$Sal=min\sqrt{tx^{2}+ty^{2}}$$

Texture Aspect Ratio (S*tr*)

The texture aspect ratio parameter (S*tr*) was also determined and used to assess the uniformity of the texture of the manufactured polymer compacts from the three polymers. S*tr* is calculated in a similar manner to S*al* as described earlier, however, S*tr* considers the minimum and the maximum radii lengths under the same conditions after applying the same threshold. A surface is reported as more uniform the closer to 1 the S*tr* value, whereas a dominant texture direction often influences the surface the closer to 0 the value is. These parameters are described in ISO 25,178 [50].

Areal Material Ratio (S*mr2*)

S*mr2* is one of a number of parameters determined from the cumulative material ratio curve. By definition, S*mr2* represents the ratio of the area of the material at the intersection line which separates the dales from the core surface to the evaluation area. Put into context, this means that S*mr2* represents the percentage of the measurement area that comprises of the deeper valley structures and is associated with the reduced valley depth (S*vk*) and pit void volume (V*vv*) parameters. S*mr2* can also be converted to calculate the percentage of ‘valleys’ that will retain lubrication. In this experimentation, this parameter is being used to determine the percentage of the compact surface available for wetting to occur for the initial hydration process. This is represented as Equation (4). For a deeper understanding of these parameters, readers are also directed to the ISO 25178 [50]:

(4) Lubrication=100−Smr2

In summary, the importance of the selected parameters used are as follow: S*al* was primarily used in this work as confirmatory parameter to determine whether the filtration of the data had been successful and that all conclusions were based on the small scale roughness of the tablet surface. S*tr* gives an indication to the uniformity of the texture on the tablet surface. S*dr* provides information on the percentage surface area gained by a tablet as a result of its texture and S*mr2* can be converted to measure lubrication which could provide an early insight into the wettability of a surface.

For this work, the 10× magnification used a 4 × 4 image area to produce the images. The vertical resolution for the 10× magnification used was 0.21 µm. The Surfstand™ software (version 6.0.0) (Taylor Hobson, Leicester, UK and University of Huddersfield, Huddersfield, UK) was used to analyse the acquired focus variation images.

#### 2.2.6. Dissolution Imaging (Whole Dosage Cell Measurement)

**Figure 2:**
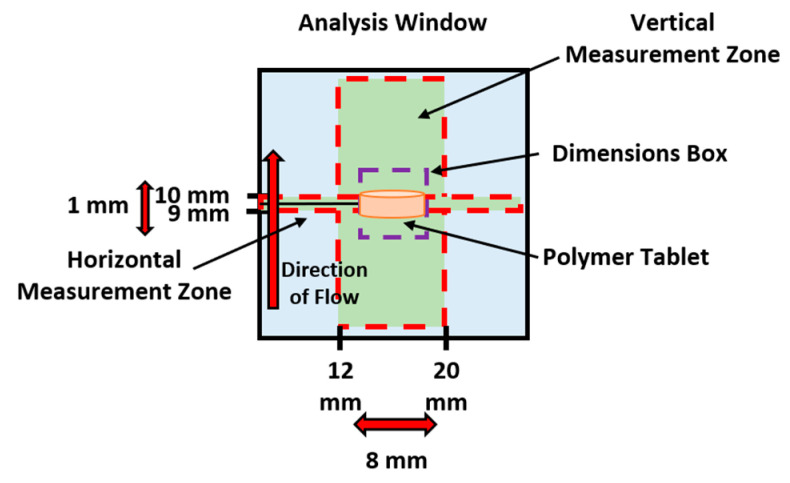
A schematic representation of the analysis conducted on the polymer compacts, displaying the measurement zones and the tablet orientation.

The methodology devised by Ward et al. [3] was followed. Briefly, all hydrophilic polymer compacts (XG, PEO, or HPMC) were analysed in the axial orientation (Figure 2) using a stainless steel wire holder. All experiments were then conducted in triplicate at 37 °C at a flow rate of 8.2 mL/min over a 2 h period. All images were recorded using the 520 nm LED. Once each experiment was completed, the experiment files were analysed using the bespoke analysis software (version 3.0.22), with the wider measurement zone and an absorbance threshold of 50 mAu. Data were exported to Microsoft Excel™ (2016 version) for data processing. The images obtained from the analysis were also adjusted to highlight the dry core and subsequent hydrated layers. Tablet growth measurements were displayed as a percentage gain based on the initial reading of tablet size (Equation (5)). Microsoft Excel was used to calculate and plot all growth data points:

(5) $$Growth\;\left(\%\right)=\left(\left(\frac{h_{t\;=\;0}+\;h_{t\;=\;x}}{h_{t\;=\;0}}\right)-1\right)\times 100$$

where *h* = tablet height, *t* = time, and *x* = a given time point.

## 3. Results and Discussion

### 3.1. Micrometric and Tabletting Properties

**Figure 3:**
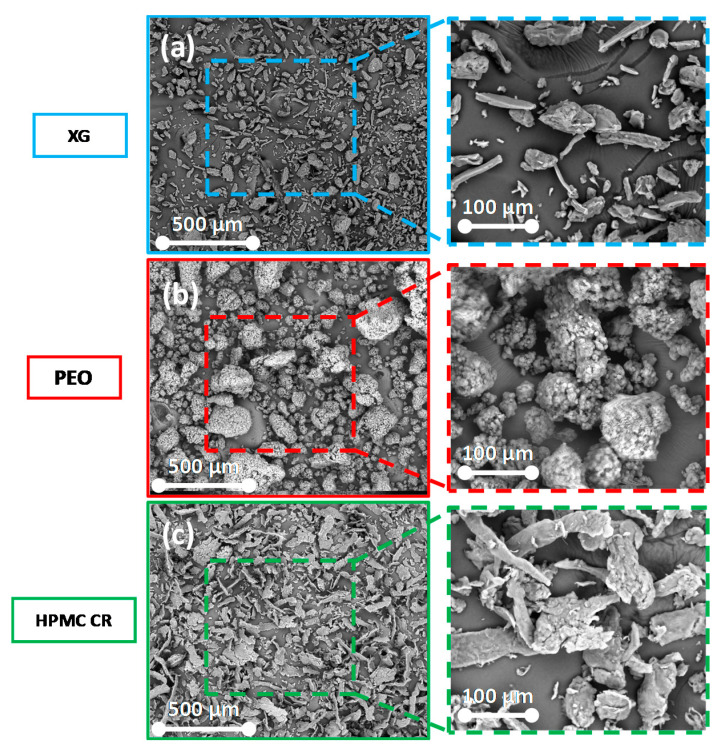
Scanning electron micrographs of the polymers tested: (**a**) xanthan gum (XG), (**b**) polyethylene oxide (PEO), (**c**) hydroxypropyl methylcellulose (HPMC CR).

XG displayed a blend of particle morphologies within the bulk powder including rod-like and irregular particles (Figure 3a). This blend of particle morphologies could be due to the extraction and manufacturer processing of the natural polymer. PEO (Figure 3b) displayed larger particles which were more rounded in nature. On closer inspection, using a higher magnification, these particles appeared to be agglomerates of smaller particles present in the bulk powder, giving the larger agglomerates a microporous structure. The HPMC CR micrographs displayed (Figure 3c) particles with a thin ribbon like morphology [53]. All the polymers have characteristics that could influence the surface topography of the tablets, and therefore, the hydration of its polymer compacts.

**Figure 4:**
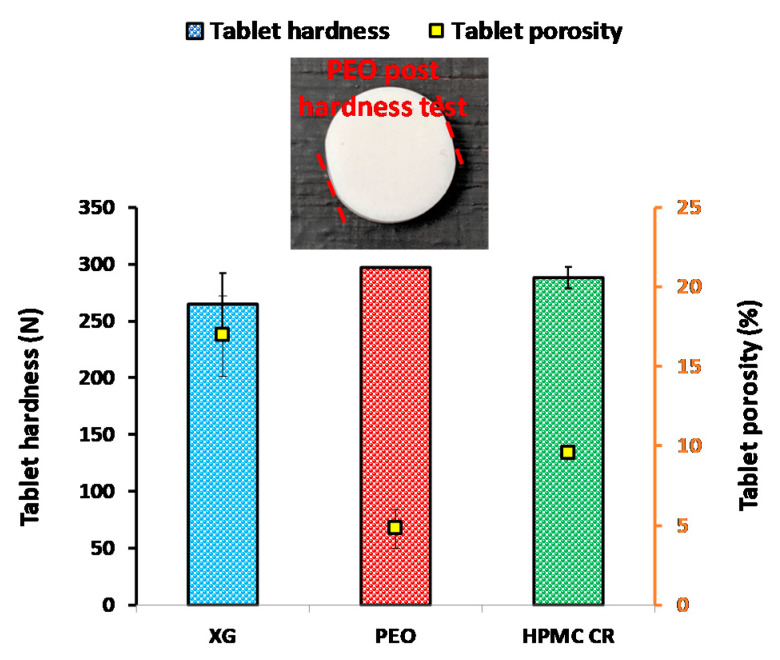
Polymer compact properties of hardness and porosity assessment for the three polymers tested. Note: XG is xanthan gum, PEO is polyethylene oxide, and HPMC CR is hypromellose K100M grade. Insert is a photograph highlighting the deformation that occurred to the polyethylene oxide (PEO) compacts during the hardness test. The red dashed lines indicate the sides of the tablet deformed during testing.

Figure 4 displays the results from the porosity and hardness measurements for the polymer compacts. XG displayed the highest porosity of all the polymers compacted at 10 kN, with a porosity of approximately 17%. From a porosity perspective, it could be assumed that the dissolution media may penetrate into the XG compacts at a faster rate than the PEO and HPMC polymer compacts, potentially leading to greater hydration and rate of swelling. XG also produced comparably softer compacts with a hardness of 265 N and with a relatively higher deviation compared to PEO and HPMC CR. PEO produced compacts that were the hardest of all the polymers tested (>297 N). The tablet did not fracture and it is therefore important to note that this value represents the upper limit of the hardness tester. It is likely that the excessive hardness was a result of the plastic nature of the polymer [11]. This is supported by the deformation that occurred to the edges of the compacts at this force (insert in Figure 4 with red dashed lines showing the deformation). This increased hardness for the PEO compacts was also supported by the lowest compact porosity for all the polymers tested (approximately 5%) (Figure 4). This observation leads to the hypothesis that the PEO compacts may potentially hydrate at a slower rate than the other polymers, resulting in a lower rate of growth of the gel layer. The HPMC CR compact produced a porosity of ~10% and a hardness of ~288 N at 10 kN (Figure 4).

### 3.2. Focus Variation Microscopy

**Figure 5:**
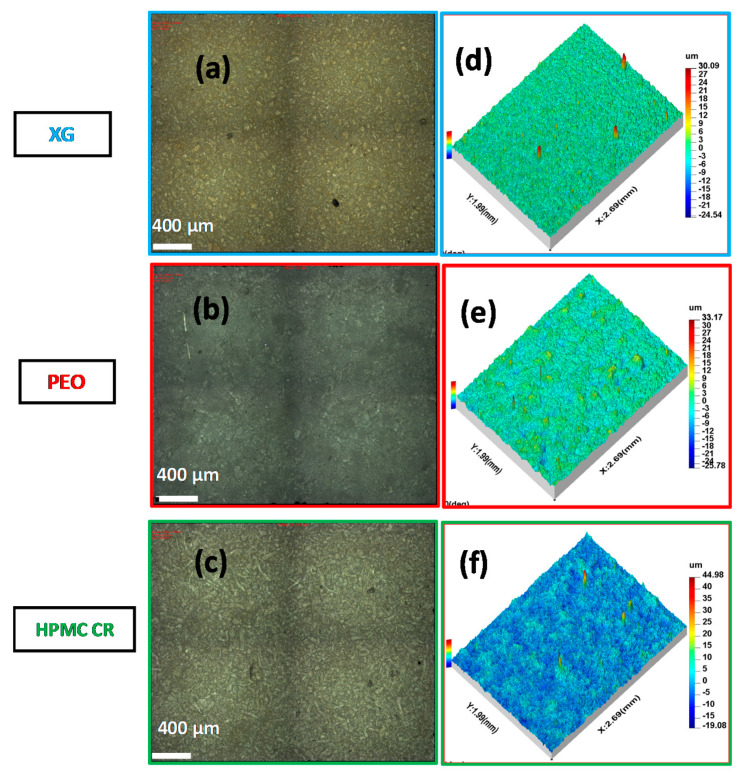
Optical images obtained from focus variation microscopy of the polymer compact surfaces at 10× magnifications: (**a**) xanthan gum (XG), (**b**) polyethylene oxide (PEO), (**c**) hydroxypropyl methylcellulose (HPMC CR). 3D roughness maps obtained from the focus variation microscope for the polymer compact surfaces at 10× magnifications: (**d**) xanthan gum (XG), (**e**) polyethylene oxide (PEO), (**f**) hydroxypropyl methylcellulose (HPMC CR).

The focus variation microscopy provided a unique insight into how the morphology of the bulk powders identified in the SEM influenced the surface topography of the polymer compacts. Images obtained from the surface assessment of the polymer compacts are shown in Figure 5a–c. These are also accompanied by 3D roughness plots displayed as Figure 5d–f. The 3D roughness plots were used to calculate the surface parameters in Table 2. All surface profiles displayed a S*al* value of below 0.1 mm. This indicated that only high frequency components (i.e., roughness) were considered and that the analysis had been successful. The presence of the smaller angular particles in the XG bulk powder can be seen across the surface of the polymer compact in Figure 5a. This can also be visualised in the 3D roughness plot where a uniform spread of small peaks can be seen across the surface (Figure 5d). The S*tr* parameter, which provides a measurement of the uniformity of the texture on the surface of the polymer compacts, indicated that the surface texture was uniform in all directions due to a value greater than 0.5 (Table 2). XG displayed a low S*dr* value of 3.63 ± 0.28% at the 10× magnification despite the texture on the surface of the compact. ISO guidelines recommend that S*dr* values are below 5% for a surface as in theory all manufactured surfaces will display some texture. An interesting observation was that XG displayed the lowest S*mr2* value of the polymers tested with a value of 89.82 ± 0.26% at the 10× magnification. The S*mr2* parameter is influenced by the deeper surface pores present across the surface and suggests that 10.18% of the surface is suitable for ‘lubricant’ retention and could provide a further indicator to a faster hydration and growth rate for the XG compacts.

The PEO polymer compact surface showed the presence of the larger spherical particulates across the surface of the compact identified by SEM. These particulates can be seen in the 3D roughness maps in Figure 5e. The optical images in Figure 5b also highlighted potential points of plastic deformation indicated by the darker grey areas across the surface. Despite the presence of the larger agglomerates, they were still distributed evenly across the compact surface, leading to an S*tr* value of 0.93 ± 0.03. The PEO compact surface also displayed the lowest S*dr* value of the three polymers tested with a value of 2.38 ± 0.11% at the 10× magnification. This was a good indicator of the plastic behaviour experienced by the polymer during compaction and correlates with the high hardness (mechanical strength) required to deform the compact. The relatively higher S*mr2* value of 90.96 ± 0.48% (at 10× magnification) indicates a lower percentage of the surface potentially suitable for ‘lubrication’ as compared to the XG compacts. The HPMC CR compacts surfaces displayed the effects of the ribbon like particles (Figure 5c). This was further supported by the 3D roughness maps (Figure 5f), which displayed the longer valleys caused by the ribbon like particulates of the CR grade. In a similar trend to the XG and PEO polymers, the HPMC CR grade displayed an S*dr* value close to 5%. The S*mr2* parameter indicated that the polymer had a surface suitable for ‘lubrication’ with a value of 91.50 ± 0.31% at the 10× magnification. The focus variation microscopy therefore provided a novel assessment of the compact surfaces for the three polymers in this study with the production of both 2D optical and 3D images. The technique also helped to reinforce the conclusions determined from both porosity and hardness measurements, with the calculation of surface parameters such as S*dr* and S*mr2*. Values from S*mr2* therefore suggests wetting, and hence, early gel formation to occur in the order of XG > PEO > HPMC CR.

### 3.3. Dissolution Imaging and Swelling Assessment

**Figure 6:**
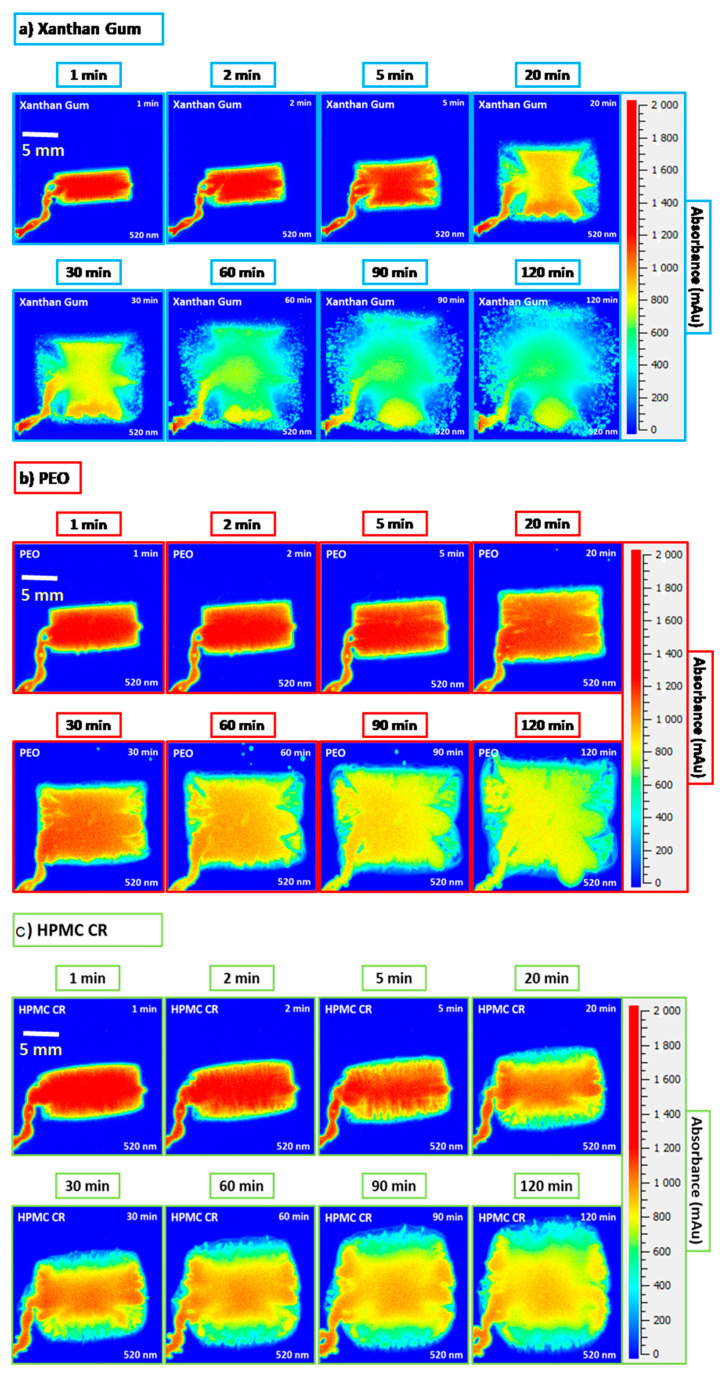
Optical images obtained from the dissolution imaging system at the 520 nm wavelength: (**a**) XG, (**b**) PEO, and (**c**) HPMC CR. Note: Images are taken from the 2 h experiment at time intervals of 1, 2, 5, 20, 30, 60, 90 and 120 min. XG is xanthan gum, PEO is polyethylene oxide, and HPMC CR is hypromellose K100M CR grade.

**Figure 7:**
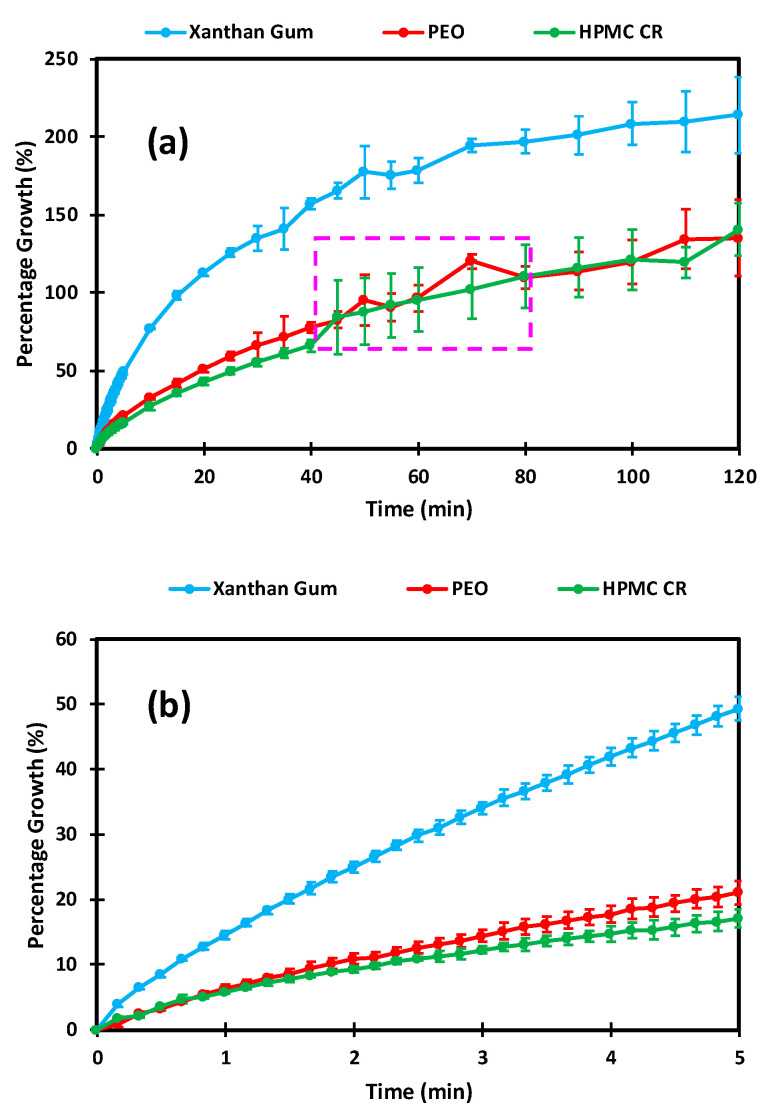
Percentage growth profiles obtained for the polymer compacts: (**a**) Percentage growth over the full 2 h experiment and (**b**) Initial percentage growth at the first 5 min of hydration. Note: The percentage growth is relative to the starting height of each polymer compact. The pink dashed box indicates deviation as a result of air bubbles for the hydroxypropyl methylcellulose (HPMC CR) compacts.

Figure 6a displays the dissolution images obtained for the XG polymer compacts. Visual assessment indicated that XG hydrated, rapidly leading to significant levels of polymer growth. The 20 min image also displays the formation of deep channels in the gel layer, potentially caused by the high porosity of the polymer compact. This observation can be particularly useful as it is likely that channels such as these could be the cause of dose dumping and formulation failure for ER therapies. As the experiment progressed from the 60 min time point onwards, the gel became increasingly translucent, indicative of complete polymer hydration. This was also reflected in the percentage growth profile displayed in Figure 7a. The profile displayed a plateau from 80 min onwards, suggesting that the growth rate had reached an equilibrium with the erosion of the gel layer.

The growth profile also displayed the extent to which XG “out swelled” the PEO and HPMC CR polymers in this study with regards to both the extent and rate of growth. By the end-point of the experiment at 120 min, the XG compacts reached an average growth percentage of 214 ± 24% which was at least 70% greater than the other polymers used in this study. The initial (first few min) hydration of XG is also displayed in Figure 7b. This graph displayed how the polymer reached an average growth of 49 ± 2% in the first 5 min of the hydration process. This supports the predictions from both the focus variation and the porosity assessment that the significant porosity of the XG compacts may lead to a faster initial hydration and consequently growth. Figure 6b displays the dissolution images for the PEO compacts. The images highlight that PEO exhibited significant gel formation and growth despite having the lowest compact porosity of all three polymers tested. When compared to the XG compacts, PEO also developed a square shaped gel layer, indicating relatively similar rates of growth in both the axial and radial directions. However, in contrast to the XG compacts, the PEO gel layer remained particularly dense, thus indicating a slower ingress of water to the tablet core. This observation is also supported by the slower development of the deeper channels in the gel layer, which appeared from the 60 min time point onwards. The dissolution images of the PEO compacts also provided further evidence for the use of colour adjustment as described by Ward et al. [3]. This allowed for the clear distinction of the various gel densities developing around the compact core to be observed. The growth profile for the PEO compacts is shown in Figure 7. Here, the PEO compacts showed no signs of plateau by the 2 h end-point. The PEO compacts displayed a similar growth rate to the HPMC CR compacts despite a significantly lower compact porosity (Figure 4) and reached an average growth percentage of 135 ± 9%. Interestingly, the PEO growth profile contradicted the hypothesis that the PEO compacts may take relatively longer to wet and hydrate than the other polymers tested in this study due to its porosity (Figure 4). This indicates that the chemistry (structure of the polymer which can affect its solubility) of the polymer may have a greater influence on gel growth than physical characteristics such as porosity. For example, it is known that the differences in the methoxyl content for the different grades of HPMC brings about a chemical and physical variation within the polymer that can affect thermal gelation as well as the self-diffusion coefficient (SDS) [16,24]. The dissolution images shown in Figure 6c showed similar behaviour to that observed by Ward et al., despite the increase in compact size from 8 to 10 mm [3]. The HPMC CR compacts developed a gel layer with a circular shape. The growth profiles in Figure 7a showed significant influence of air bubbles on the HPMC CR growth profile (indicated by the spike in the pink dashed box of Figure 7a). The initial hydration profiles displayed in Figure 7b also highlight a difference in the initial hydration between the polymers tested. The HPMC CR polymer experienced an initial hydration, with a total percentage growth of 17 ± 1% which was the lowest of the three polymers after the 5 min time point. This observation was thus in keeping with the hypothesis from the focus variation microscope that the initial hydration of the polymers tested may be in the order of XG > PEO > HPMC CR due to the surfaces available for initial hydration calculated from the S*mr2* parameter.

## 4. Conclusions

This work represents a development and validation of the method developed by Ward et al. [3] in testing the method specifics against three different hydrophilic polymers representing the different classifications of hydrophilic polymers. This work also introduces the use of focus variation microscopy to profile the surface topography of polymer compacts post compaction and attempts to attribute differences in surface topography to dissolution behaviour. All three polymers tested were successfully profiled and displayed significantly different physical properties when compacted at equivalent force and mass. XG produced compacts with the highest compact porosity, whilst the PEO produced compacts with the highest compact hardness. Focus variation microscopy provided a deeper insight into the influence of particle morphology on surface roughness. The S*mr2* parameter indicating the surface available for lubrication suggested the surface available for wetting to be in the order of XG > PEO > HPMC CR, thus predicting XG to wet and swell the fastest. The dissolution imaging instrument successfully profiled all three polymers using the developed methodology and was found to be capable in distinguishing the subtle nuances between the hydration of all the polymers. Dissolution imaging found the XG compacts to swell rapidly and at a greater rate than the other polymers tested with the swelling data, also indicating that initial wetting and swelling did indeed follow the order of XG >PEO > HPMC CR. The images produced also provided a novel insight into the growth and development of gels by imaging features such as deep channels, density differences, and air bubbles. Overall, this work represents a novel investigation into the assessment and analysis of three commonly used hydrophilic polymers using two novel imaging techniques to provide a rapid assessment of pharmaceutical materials to provide an in-depth insight to a formulator.

## Figures and Tables

**Table 1:** A summary of the ISO guidelines governing profile and areal measurement of surfaces [50].

Profile Standards	Areal Standards (ISO 25178)
Nominal characteristics of contact (stylus) instruments. (ISO 3274)	Part 1: Areal surface texture drawing indications.
Rules and procedures for the assessment of surface texture (ISO 4288)	Part 2: Terms, definitions and surface texture parameters.
Metrological characteristics of phase correct filters (ISO 11562)	Part 3: Specification operators.
Motif Parameters (ISO 12085)	Part 4: Comparison rules
Surfaces having stratified functional properties—Part 1 (ISO 13565 part 1)	Part 5: Verification Operators
Surfaces having stratified functional properties—Part 2 (ISO 13565 part 2)	Part 6: Classification of methods for measuring surface texture.
Terms definitions and surface texture parameters (ISO 4287)	Part 601: Nominal characteristics of contact stylus instruments
Measurement Standards—material measures (ISO 5436 part 1)	Part 602: Nominal characteristics of non-contact (confocal chromatic probe) instruments.
Software measurement standards (ISO 5436 part 2)	Part 603: Nominal characteristics of non-contact (phase shifting interferometric microscopy) instruments.
Calibration of contact stylus instruments (ISO 12179)	Part 604: Nominal characteristics of non-contact (coherence scanning interferometry) instruments.
Surfaces having stratified functional properties—Part 3 (ISO 13565 part 3)	Part 605: Nominal characteristics of non-contact (point autofocus) instruments
Indication of surface texture in technical product documentation (ISO 1302)	Part 606: Nominal characteristics of non-contact (variable focus) instruments

**Table 2:** Table displaying average surface parameter values at 10× magnification for the three hydrophilic polymers.

Surface Parameters			
10× Magnification			
Parameter	Xanthan Gum (XG)	Polyethylene oxide (PEO)	Hydroxypropyl methylcellulose (HPMC CR)
S*tr*	0.96 ± 0.26	0.93 ± 0.03	0.90 ± 0.04
S*al* (mm)	0.03 ± 0.02	0.07 ± 0.01	0.06 ± 0.00
S*dr* (%)	3.63 ± 0.28	2.38 ± 0.11	4.38 ± 0.33
S*mr2* (%)	89.82 ± 0.26	90.96 ± 0.48	91.50 ± 0.31
